# Circularly polarised luminescence in an RNA-based homochiral, self-repairing, coordination polymer hydrogel†

**DOI:** 10.1039/d2tc00366j

**Published:** 2022-04-20

**Authors:** Osama El-Zubir, Pablo Rojas Martinez, Gema Dura, Lamia L.G. Al-Mahamad, Thomas Pope, Thomas J. Penfold, Lewis E. Mackenzie, Robert Pal, Jackie Mosely, Fabio Cucinotta, Liam F. McGarry, Benjamin R. Horrocks, Andrew Houlton

**Affiliations:** Chemical Nanoscience Labs, Chemistry, School of Natural Sciences, Newcastle University Newcastle upon Tyne NE1 7RU UK andrew.houlton@ncl.ac.uk; Departamento de Química Inorgánica, Orgánica y Bioquímica, Facultad de Ciencias y Tecnologías Químicas, UCLM Spain; Department of Chemistry, College of Science, Mustansiriyah University Baghdad Iraq; Department of Chemistry, Durham University South Road Durham DH1 3LE UK

## Abstract

The aqueous equimolar reaction of Ag(i) ions with the thionucleoside enantiomer (−)6-thioguanosine, ((−)6tGH), yields a one-dimensional coordination polymer {Ag(−)tG}_*n*_, the self-assembly of which generates left-handed helical chains. The resulting helicity induces an enhanced chiro-optical response compared to the parent ligand. DFT calculations indicate that this enhancement is due to delocalisation of the excited state along the helical chains, with 7 units being required to converge the calculated CD spectra. At concentrations ≥15 mmol l^−1^ reactions form a sample-spanning hydrogel which shows self-repair capabilities with instantaneous recovery in which the dynamic reversibility of the coordination chains appears to play a role. The resulting gel exhibits circularly polarised luminescence (CPL) with a large dissymmetry factor of −0.07 ± 0.01 at 735 nm, a phenomenon not previously observed for this class of coordination polymer.

## Abstract 2

The aqueous equimolar reaction of Ag(i) ions with the thionucleoside enantiomer (−)6-thioguanosine, ((−)6tGH), yields a one-dimensional coordination polymer {Ag(−)tG}_*n*_, the self-assembly of which generates left-handed helical chains that display CPL.

## Graphical abstract

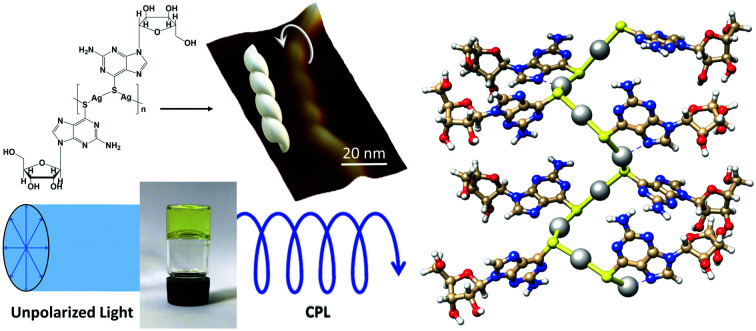

## Introduction

The transformative impact of molecular-based compounds in technologies such as OLED displays and lighting,^1–4^ security tags,^5^ and optical quantum memory,^6–8^ has significantly increased the interest in materials exhibiting exceptional or novel optical properties, such as polarization.^9–11^ Traditionally, circularly polarized light is generated from unpolarized light using a combination of linear polarizers and quarter-wave plates. However, such indirect methods are inefficient leading to 50% loss of the energy during this transformation, motivating a significant research effort into searching for, and designing new types of molecules and materials that can directly generate circularly polarized (CP) light.^12^

One of the challenges in this field is achieving a significant dissymmetry, *i.e.* the selectivity for left-handed *versus* right-handed CP light. This arises because the dissymmetry is sensitive to both the magnitude and relative orientations of the magnetic and electric transition dipoles within the chromophore. However, the majority of molecules designed to absorb or emit light, exhibit dipole-allowed electronic transitions and the magnetic transition dipole moment (***m***) is overwhelmed by the electric transition dipole moment (***μ***), resulting in very small dissymmetry or so called *g*-factors, which are usually <10^−2^.^13^ The origin of this is the electric dipole approximation, which assumes the wavelength of light is much larger than the typical size of a molecule.

Recently polymers have attracted increasing attention^14^ as potential candidates for producing large dissymmetry factors. Furthermore, the ability to prepare such materials by self-assembly offers additional advantages compared to more traditional synthetic methods as demonstrated, particularly for organic systems, with supramolecular chirality^15^ and the control of helical handedness.^16,17^

Using helical metallopolymers, Nitschke and co-workers^16^ recently demonstrated the influence of self-assembly on the dissymmetric response of a material. In the disassembled state the monomers exhibit no CD response, due to the remoteness of the chiral centres. However, upon self-assembly, a double helix structure was formed giving rise to a CD response which increased as a function of polymer length. This was accompanied by a red-shift in the absorption spectrum and was therefore associated with delocalisation of the excited state over the polymer.

Coinage-metal thiolate (CMT) coordination polymers are a class of self-assembled compounds^18^ that find use in medicine, as anti-arthritic^19^ and antimicrobial agents,^20^ as models of metal–thiolate monolayers (SAMs)^21^ and display a range of useful physicochemical properties, such as electrical conductivity,^22,23^ and tunable luminescence.^24,25^ These materials predominantly feature one-dimensional (1D) coordination chain structures, as determined by single crystal and powder X-ray diffraction.^18^ However, while some of these chains are chiral the materials generally crystallise in centrosymmetric^25,26^ or racemic^27^ arrangements on account of left and right-handed helices forming equally. Furthermore, these compounds are typically highly insoluble making low-temperature solution processing difficult.^28^ However, we have recently extended the scope of this class of material showing the thionucleoside, (−)6-thioguanosine ((−)6-tGH), to assemble helical coordination chains upon reaction with Au(i) ions, in the form of a readily processable hydrogel.^29^ The large chiro-optics of the resulting gel suggested a preferential handedness to the polymer chain assembly, though this was not confirmed.

We reasoned that if this assembly mechanism for univalent coinage-metals ions is general, then it could provide CMT materials exhibiting large circularly polarized luminescence (CPL) with high luminescence dissymmetry factor (*g*_lum_) values.^9^ Here we demonstrate that this is indeed the case with the reaction of (−)6-tGH and Ag(i) ions which spontaneously forms {Ag(i)-(−)6tG}_*n*_, 1, as a luminescent self-healing hydrogel. Individual coordination chains, microns in length, adopt a purely left-handed helical arrangement, as determined by AFM, CD and DFT calculations. At higher concentrations these further assemble to produce a gel with much enhanced chiro-optics compared to the parent ligand. The homochiral helical macromolecular structure, combined with the intrinsic luminescence of the Ag-thiolate chain, result in a material that exhibits CPL with *g*_lum_ = −0.07 ± 0.01; the large dissymmetry factor generated is comparable to circularly polarised luminescence generated by lanthanide complexes,^30^ and is orders of magnitude greater than other classes of chiral emitters such as helicines, ketones, comparable coordination-based systems^31–35^ and is competitive with helical polymers in general.^17,36,37^

## Results and discussion

### Ag(i)-thioguanosine preparation and characterization of homochiral helical chains

**Fig. 1:**
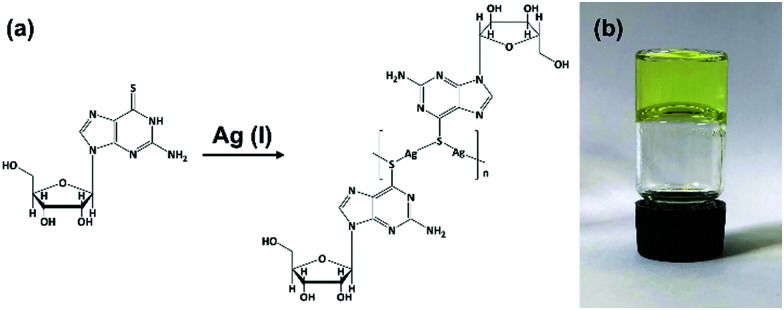
Reaction scheme for forming Ag–thioguanosine, 1. (a) Proposed structure for Ag(i):6-thioguanosine, **1**, and (b) an optical image of the yellow hydrogel **1** inverted in a glass vial.

Equimolar reactions of an aqueous dispersion of 6-tGH with AgNO_3_ lead to a change from colourless to pale yellow and formation of a product characterized as the one-dimensional coordination polymer 1 (Fig. 1). This is indicated in the UV-visible spectrum (Supplementary Fig. S1a, ESI†) by a broadening of the longer wavelength >300 nm band which extends further into the red in keeping with thiolate–metal bonding. Changes to the FTIR spectrum of 1 are similarly consistent with this assignment. Upon complex formation the intense stretch at 1207 cm^−1^ attributed to C=S thione is reduced significantly and shifted (1192 cm^−1^) (Supplementary Fig. S2, ESI†). The polymeric nature of 1 is supported by MALDI-MS analysis (Supplementary Fig. S3, ESI†) with peaks corresponding to oligomeric species as: [Ag_5_L_4_]^+^ (found (*m*/*z*) 1730.9; calc. 1730.8) [Ag_4_L_3_]^+^ (found (*m*/*z*) 1326.0; calc (*m*/*z*) 1325.8), [Ag_3_L_2_]^+^ (found (*m*/*z*) 921.2; calc. 920.8). [L corresponds to a deprotonated, anionic form of 6-thioguanosine [C_10_H_12_N_5_O_4_S]^−^].

CMT coordination polymers are known to show luminescence,^18,24,29^ and this is indeed the case for 1, though an unusual concentration dependence was observed (see below). In aqueous solution, 1 exhibits room temperature luminescence that is minimally shifted compared to the free ligand (6tG: *λ*_Ex_ = 350 nm; *λ*_Em_ = 418 nm; 1: *λ*_Ex_ = 350 nm; *λ*_Em_ = 414 nm) suggesting that the emitting state(s) is predominantly localized on the organic ligand. However, the effect of metal binding is seen in the marked change in the emission lifetime (Supplementary Fig. S4, ESI†) with a longer lifetime in 1 (7.00 ns) compared to the free ligand (0.40 ns). This increase can be ascribed to the breakdown of the tautomer equilibrium^38,39^ of 6TG upon coordination to Ag(i) in its thioenolic form. The polymerisation would also make the system more rigid leading to a reduction of the non-radiative decay rate.

**Fig. 2:**
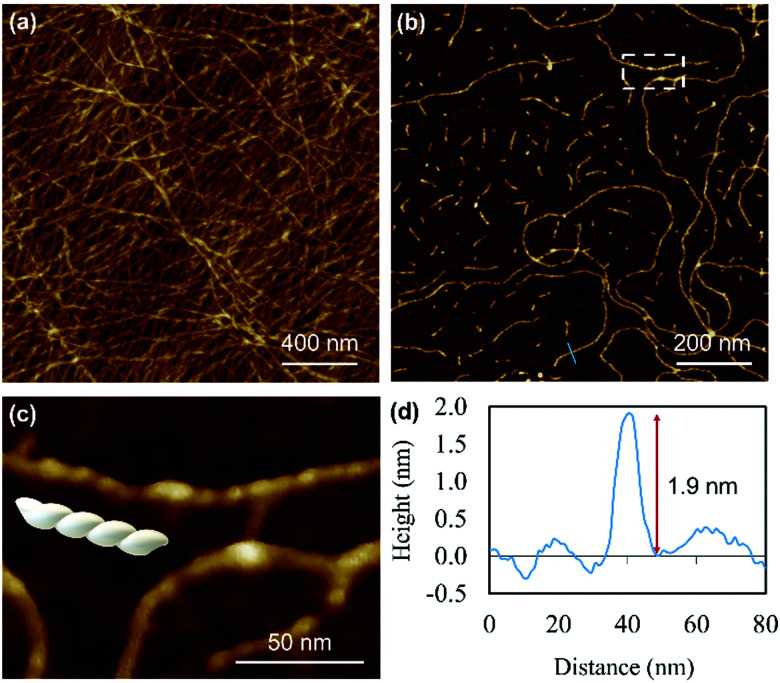
AFM images of Ag–thioguanosine xerogel drop-cast onto a silicon wafer. (a) AFM height images of an Ag-6tG xerogel thin film. (b) AFM image shows the molecular chains of the Ag–tG. (c) A zoom area of the AFM image shows the helical structure of individual molecular chains. The cross-section (d) along the blue line in (b) shows the height of an individual chain.

**Fig. 3:**
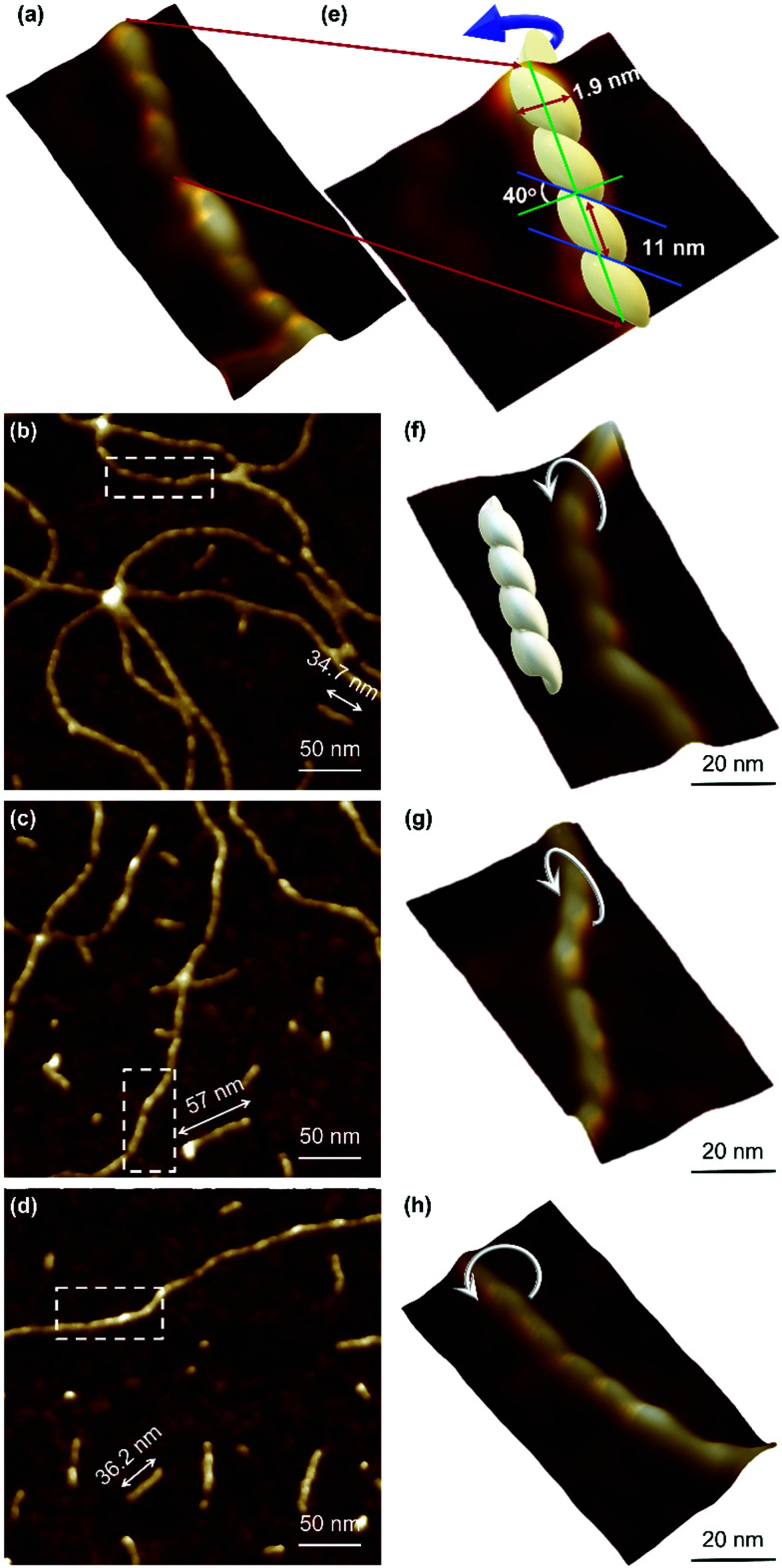
AFM height images of Ag–thioguanosine xerogel. (a) 3D section shows the helical structure of a polymer chain. (e) A model has been built on the top part of the 3D structure in (a) to show the helicity and the periodicity of the polymer. (b)–(d) High resolution AFM images show the helical structure of individual molecular chains. (f)–(h) 3D AFM height images of part of the single helical structure contained in the white boxes in the images (b)–(d).

Atomic force microscopy (AFM) confirmed the one-dimensional polymeric nature of 1 with strands assembling into the entangled matrix seen in Fig. 2(a). High resolution, small scan-range, imaging of the sample reveals chains with heights of 1.86 ± 0.24 nm, Fig. 2. Furthermore, the 1D coordination polymer chains can be seen to be helical and are exclusively of a single, left-handed, sense (Fig. 2 and 3). In Fig. 3 AFM height images of representative individual strands are shown in detail and reveal a periodicity of 11.21 ± 0.99 nm and helical lead angle 40°. The pitch and diameter of the helix revealed by AFM is not necessarily expected to match the molecular dimensions (below) owing to molecule–substrate interactions and tip convolution effects. Nevertheless, we reason that the homochirality of the coordination chains is attributed to the intrinsic chirality of the ligand directing the sense of the metal–ligand bond forming self-assembly.

**Fig. 4:**
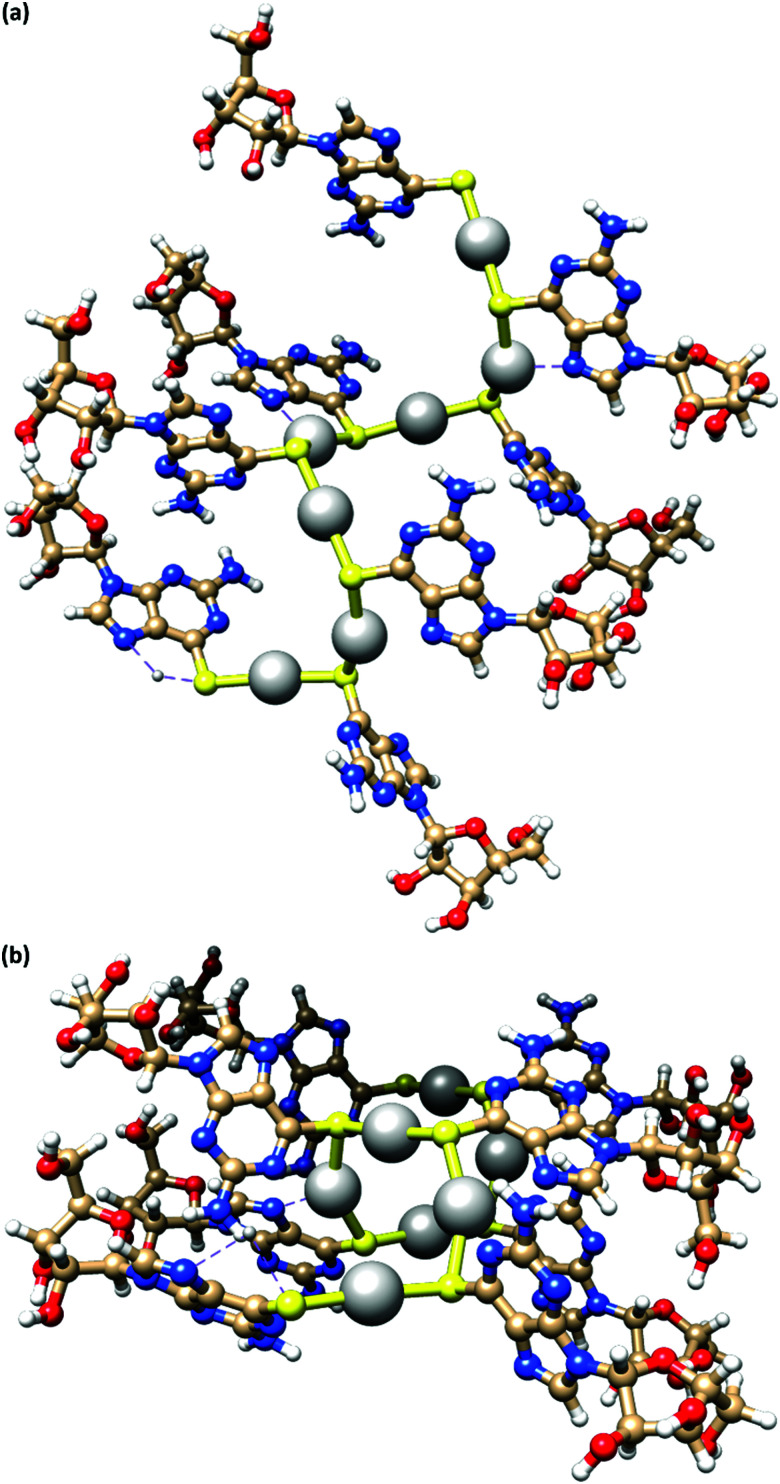
A model of the coordination polymer chain of **1**, viewed onto (a) and down (b) the helical axis of 1D supramolecular structure.

Fig. 4 depicts a DFT geometry-optimized model of 1 derived from crystallographically characterized Ag(i)–thiolate polymers and consistent with the above data.^18,20^ The structure features a left-handed helical Ag(i)–thiolate chain as the main backbone of the polymer. The molecular chain has a diameter ∼2.1 nm, in reasonable agreement with the height of the smallest strands seen by AFM (see Fig. 2(d)) confirming these to be single or, at most, entwined duplex^20^ strands. The coordination geometry at silver is essentially linear (<S–Ag–S 166–175°) and neighbouring Ag⋯Ag distances lie in the range 3.36–3.73 Å.

The helical pitch along the chain of this model is 8.5 Å and, as a consequence, there is an absence of intra-strand nucleobase stacking due to this large inter-base separation. The structure arranges the ribose groups to project out from the main coordination chain; this maximizes hydrogen bonding interactions with solvent water molecules as required for gel formation (see below).

The X-ray diffraction data from 1 as xerogel shows broad peaks indicating that the sample was amorphous, (Supplementary Fig. S5, ESI†). Data analysis employed a simplified Rietveld method by fitting a regression model comprising a sum of 5 Gaussian functions. The dominant peak is at ∼30 degrees and corresponds to distance *d* = 2*θ*/*Q* of 3.04 Å. We assign this to Ag⋯Ag distances by analogy with known Ag–thiolate polymers in the solid-state.^18^

### Circular dichroism

**Fig. 5:**
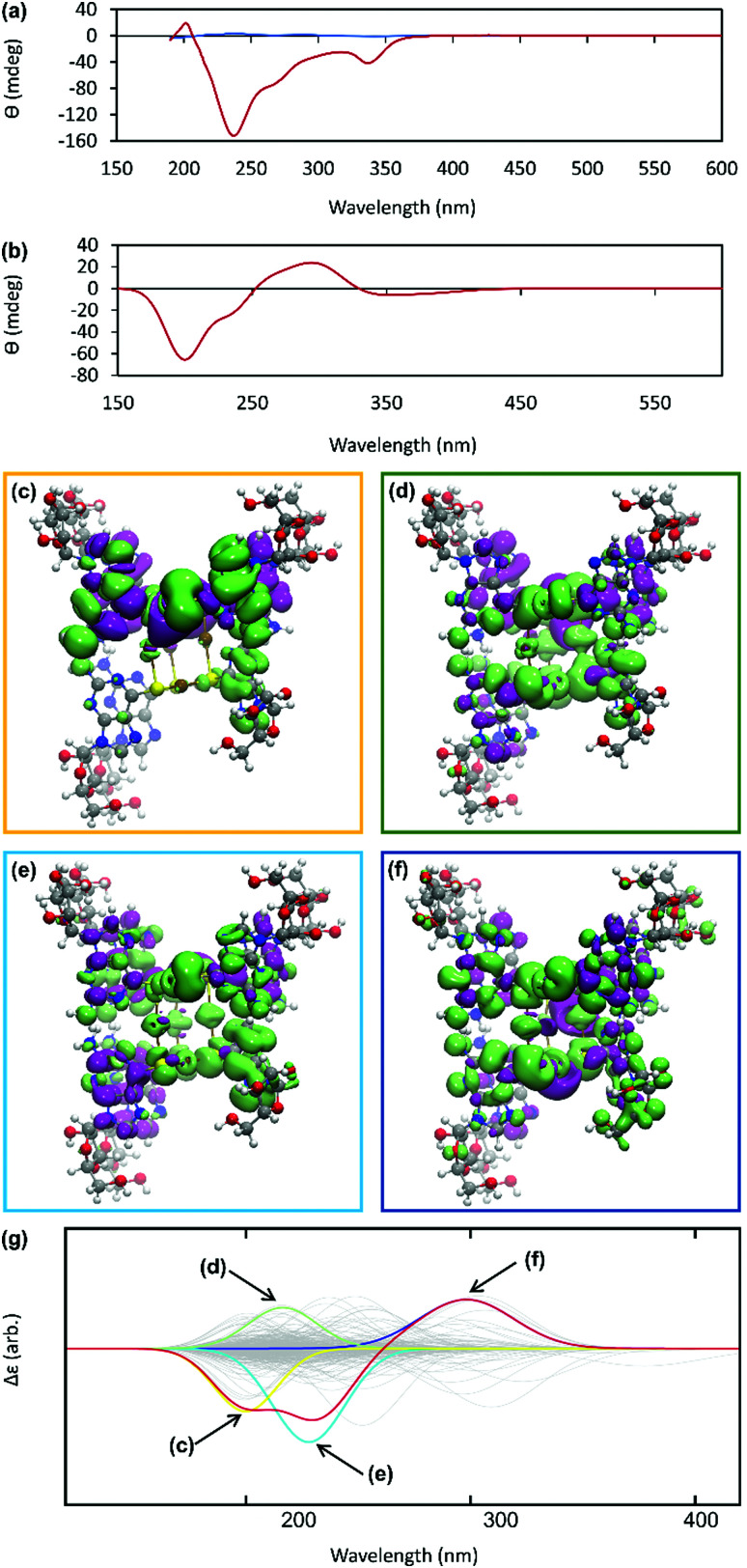
(a) CD spectrum of an aqueous solutions of 6-tGH nucleoside (blue) and of Ag–thioguanosine at a concentration of 10 mmol l^−1^ (red). (b) The circular dichroism spectrum calculated for a 7 unit oligomeric structure. (c)–(f) TDDFT(PBEO) density differences for the excitations respectively outlined in Table 1, where purple corresponds to a positive change in density and green a negative. (g) The circular dichroism reconstructed for the 7 unit oligomer using only the four dominant excitations shown above. The relevant Gaussians are highlighted and colour coded and all unused Gaussians are shown in grey.

The helicity of the molecular chains observed in 1 by AFM is also evident in the CD spectra (Fig. 5(a) and Supplementary Fig. S6, ESI†). The CD bands show a >15-fold enhancement in intensity compared to 6tGH which, typically for individual nucleosides, features bands of low intensity due to the relative remoteness of the chiral ribose-C1′ centre from the aromatic, nucleobase chromophore. Such a large enhancement in the chiro-optics is, in part, attributed to the polymeric structure but, also importantly, to the assembly of the coordination chain being directed to a single, left-handed, helical enantiomorph by the chiral ligand – a known phenomenon in other metal–oligomer systems.^16^

To better understand the CD spectra, we performed TD-DFT calculations on structures of increasing length. In these calculations, each unit contains a Ag(i) ion and the associated organic ligand in a left-handed helix. We find that after 7 units, the CD spectrum exhibits a converged line-shape with respect to the number of repeat units (Supplementary Fig. S7, ESI†). This spectrum (Fig. 5(b)) shows two pronounced dips, at 200 nm and 230 nm, which are similar to the features in the experimental spectrum (Fig. 5(a)). The calculated spectrum shows a positive peak at about 300 nm in the region where the experimental spectrum shows a dip. In this case the angle between the electric and magnetic transition dipoles is about 75°. The optical rotatory strength is given by the dot product of these two vectors and a small change in orientation can cause the angle to exceed 90° and the CD signal will change sign. This is a noted problem in the prediction of rotatory strengths by TD-DFT.^40^ The shape of the spectrum is dominated by four excitations (shown in Fig. 5(g)), summarized in Supplementary Table S1 (ESI†). The density differences associated with these transitions (Δ*ρ* = *ρ*_ES_ − *ρ*_GS_) are displayed in Fig. 5(c)–(f) and demonstrate delocalisation of the excited state (ES) over multiple units. Such delocalisation contributes to enhancement of the chiro-optical response.^41^

### Self-healing hydrogel formation and circularly polarised luminescence (CPL)

Reactions at concentrations ≥15 mmol l^−1^ spontaneously yield a sample-spanning gel that is stable to inversion tube testing. For the hydrogel, changes to the UV-visible spectrum are also apparent between these and more dilute reactions with the former showing an increase in the absorption band edge to 450 nm consistent with delocalization of the excited state (Supplementary Fig. S1b, ESI†).

Rheological measurements using oscillatory sweep tests confirmed 1 as a true gel rather than a viscous liquid as the value of the storage modulus (*G*′) is higher than the loss modulus (*G*′′), with a stiffness of 58 Pa (30 mmol l^−1^ polymer content in Supplementary Fig. S8, ESI†). Frequency sweep experiments showed *G*′ was dominant across the range of frequencies studied (0.1–100 rad s^−1^), indicating the elastic nature of the gel. The linear viscoelastic region of the gel was evaluated using oscillatory measurements and *G*′ was observed to be independent of strain up to 6% of strain applied (Supplementary Fig. S8c, ESI†). This indicates that the structure of the gels remains intact. The critical strain was found to be 100%, at which point *G*′ = *G*′′, suggesting the sample is flexible, like other supramolecular silver gels.^42,43^ The viscosity of the hydrogel, studied by shear rate experiments, showed a shear-thinning behaviour with the viscosity reducing by two orders of magnitude. Interestingly, the gel showed the capacity for self-repair as the backward experiment when the network is disrupted by shear shows rapid recovery (Supplementary Fig. S8d, ESI†). This can be ascribed to the non-covalent interaction between the strands of the higher-order assemblies of the gel, though there is also indication of dynamic reversibility to the length of strands suggesting some reversibility to the metal–ligand bonding within the chains. This is supported by AFM which, in addition to long strands, also shows some much shorter chains (between 13.8 and 60.9 nm) as evidence of this (Fig. 3).

**Fig. 6:**
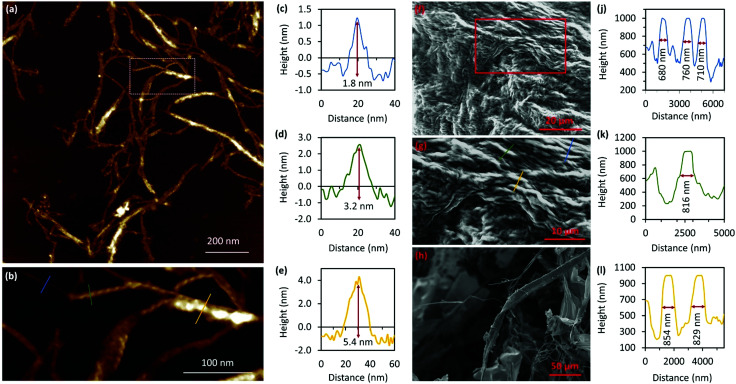
AFM and SEM images of a Ag–thioguanosine xerogel drop-cast onto a silicon wafer. Large-scan area (a) of AFM height image of the Ag-6TG xerogel. (b) A zoom area of the AFM image (a) shows individual molecular, and helical, bundle structures of multi-molecular chains. Associated cross-sections along the blue (c), green (d) and yellow (e) lines in the AFM image (b) are shown. (f) SEM images of Ag–thioguanosine xerogel film showing the microscopic metallo–thiolate structures entangled to form the gelating network. (g) A zoom area of the SEM image (f) shows the entangled fibre structure of the xerogel. (h) SEM image of freeze-dried solution of **1** at a concentration of 6 mmol^−1^. Also shown are the associated cross-sections along the blue (i), green (j) and yellow (k) lines in the SEM image (g).

**Fig. 7:**
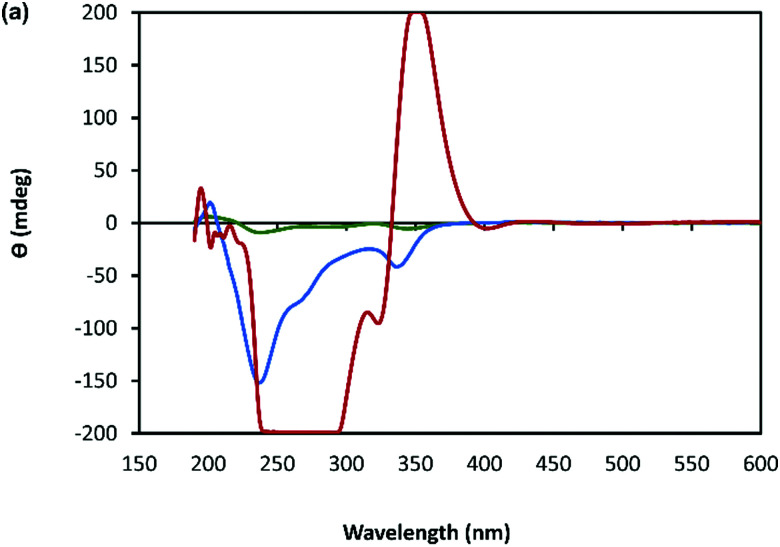
CD spectrum of a solution of Ag–thioguanosine at concentrations of 1 mmol l^−1^ (green), 10 mmol l^−1^ (blue) and 30 mmol l^−1^ (red).

A combination of AFM and electron microscopy imaging provide insight into the higher structural assembly of the gel matrix. AFM analysis of the corresponding xerogel (Fig. 6(a) and (b)) shows the entanglement of strands necessary to form the porous network for solvent trapping. Further, the appearance of twisted fibres is seen which maintain the same handedness as the individual molecular strands and demonstrates a well-ordered hierarchical assembly process. Scanning and transmission electron microscopies show a dense body of fibres (Fig. 6(f)-I and Supplementary Fig. S9, S10, ESI†) which range *ca.* 600–800 nm in diameter and are many microns in length. As expected, these larger bundles show the appearance of helicity which explains the massively enhanced chiro-optics of the hydrogel seen for 30 mmol l^−1^ samples which exceed the dynamic range of the spectrometer and which confirms the maintenance of the helicity in the higher-order assembly process of gel formation (Fig. 7 and Supplementary Fig. S6, ESI†).

Another change observed in the gel state is that 1 exhibits a new broad, red-shifted luminescence band at ∼550 nm (*λ*_Ex_ = 430 nm) (Supplementary Fig. S11b, ESI†). This is consistent with approximately head-to-tail exciton coupling between closely spaced fluorophores. The self-assembly process in the gels then involves close interaction of the aromatic nucleobase groups. In an individual polymer strand these are too far apart to interact effectively (base⋯base distances between adjacent groups are 8.5 Å, see above). However, a feature of these types of chains is the formation of entwined duplex structures^20,25,26,28^ involving metallophillic and/or stacking interactions.^44^ We expect that these, along with associated H-bonding, are major features in the final assembly.

**Fig. 8:**
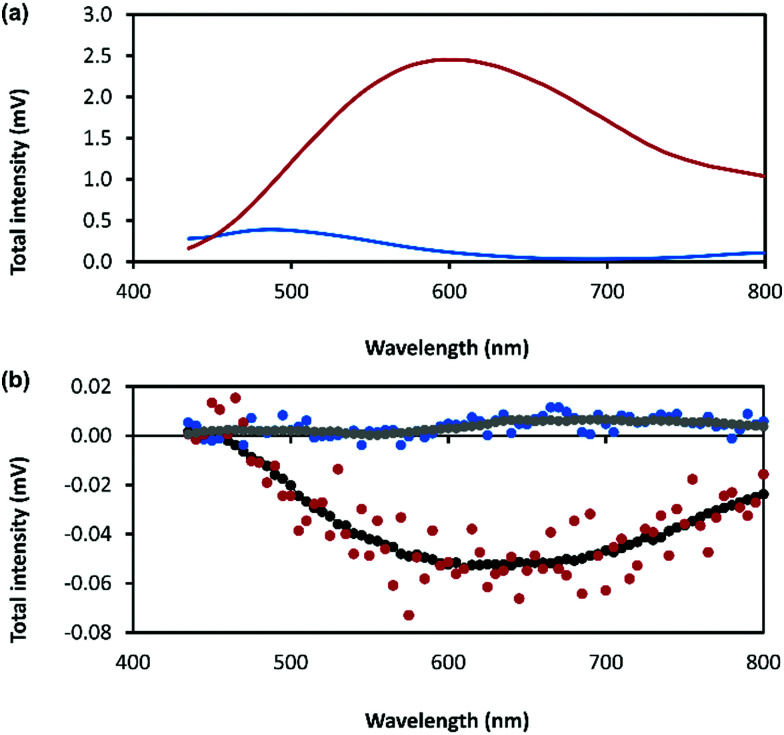
(a) Total photoluminescence spectra of 6-thioguanosine at a concentration of 30 mmol l^−1^ in 0.1 mol l^−1^ of NaOH (blue) and Ag–thioguanosine hydrogel at concentration of 30 mmol l^−1^ (red). (b) CPL raw spectra of 6 independent measurements for the 6-thioguanosine (blue), the hydrogel (red), the smoothed spectrum of the 6-thioguanosine (gray) and the smoothed spectrum of the hydrogel (black).

The combination of preferential handedness of the coordination polymer chains, their assembly into higher-order chiral structures, along with an intrinsic luminescence are ideal criteria for the material to exhibit CPL. The extent of chiral dissymmetry in fluorescence is evaluated by the luminescence dissymmetry factor (*g*_lum_), which is given by the equation *g*_lum_ = 2(*I*_L_ − *I*_R_)/(*I*_L_ + *I*_R_), where *I*_L_ and *I*_R_ refer to the intensities of the left- and right-handed circularly polarized emissions, respectively with a maximum possible *g*_lum_ value of +2/−2 for ideal left and right CPL.^45^ The luminescence dissymmetry factor (*g*_lum_) for 1 as a hydrogel (30 mmol l^−1^) is −0.04 ± 0.02 at 600 nm and −0.07 ± 0.01 at 735 nm (Fig. 8). By comparison, a solution of 6-thioguanosine (30 mmol l^−1^ in 0.1 mol l^−1^ of NaOH) shows no significant CPL. Interestingly, samples of 1 when diluted into solution also do not exhibit significant CPL (Supplementary Fig. S12, ESI†). The high CPL emission of the hydrogel can be explained by a combination of the delocalization of the excited state and the rigid self-assembled structure.^41^ A contribution from chiral scattering effects owing to the handedness of the fibrous structures of the gel is also possible.

## Conclusions

Synthetically amenable routes to new materials with useful optical properties are highly sought and self-assembly with^17^ or without hierarchical features offers such an approach. Here, we have shown that the thio-nucleoside, (−)6-thioguanosine, reacts with silver ions in a simple aqueous room temperature procedure to give the corresponding CMT-coordination polymer in the form of a self-repairing hydrogel, not as more typical, insoluble, solid. Furthermore, the self-assembly mechanism is such that only a single enantiomorphic form of the helical coordination chain is generated, which is attributed to the inherent chirality of the nucleoside ligand. The resulting homochirality, hierarchical assembly and intrinsic luminescence of the Ag–thiolate motif, provides a CMT material that displays CPL with a large dissymmetry factor of −0.07 ± 0.01 at 735 nm. Finally, as we have previously demonstrated for gold ions, this type of coordination motif can be integrated into modified oligonucleotides.^29^ We anticipate that silver analogues may be similarly synthesised and so provide new features to integrate into the important class of DNA-based materials.

## Conflicts of interest

There are no conflicts to declare.

## Supplementary Material

TC-010-D2TC00366J-s001
