# Supplementary material for: Circularly polarised luminescence in an RNA-based homochiral, self-repairing, coordination polymer hydrogel
Source: J Mater Chem C Mater. 2022 Apr 20;10(18):7329–35. doi: 10.1039/d2tc00366j (PMC9097859; doi:10.1039/d2tc00366j)
Supplement: TC-010-D2TC00366J-s001 [file TC-010-D2TC00366J-s001.pdf]

Electronic Supplementary Material (ESI) for Journal of Materials Chemistry C.
This journal is © The Royal Society of Chemistry 2022

Circularly polarised luminescence in a homochiral, self-repairing, coordination polymer hydrogel

Osama El-Zubir,[a] Pablo Rojas Martinez,[a] Gema Dura,[ab] Lamia L.G. Al-Mahamad,[c] Thomas Pope,[a] Thomas J. Penfold ,[a] Lewis E. Mackenzie,[d] Robert Pal,[d] Jackie Mosely,[d] Fabio Cucinotta,[a] Liam F. McGarry,[a] Benjamin R. Horrocks[a] and Andrew Houlton *[a]

[a] *Chemical Nanoscience Labs, Chemistry, School of Natural Sciences, Newcastle University, Newcastle upon Tyne, NE1 7RU, U.K.*
[b] *Departamento de Química Inorgánica, Orgánica y Bioquímica, Facultad de Ciencias y Tecnologías Químicas, UCLM, Spain.*
[c] *Department of Chemistry, College of Science, Mustansiriya University, Baghdad, Iraq.*
[d] *Department of Chemistry, Durham University, South Road, Durham, DH1 3LE, UK.*
[*] *Corresponding author. Email: andrew.houlton@ncl.ac.uk.*

Supplementary Information

Table of Contents

METHODS 2
- MATERIALS. 2
- PREPARATION OF 1. 2
- ATOMIC FORCE MICROSCOPY (AFM) 2
- ULTRAVIOLET-VISIBLE SPECTROSCOPY (UV-VIS) 2
- FTIR SPECTROSCOPY. 2
- MATRIX-ASSISTED LASER DESORPTION/IONIZATION SPECTROMETRY (MALDI-TOF) 2
- POWDER X-RAY DIFFRACTION (XRD) 2
- FLUORESCENCE SPECTROSCOPY. 2
- CIRCULAR DICHROISM. 3
- COMPUTATIONAL DETAILS. 3
- RHEOLOGY. 3
- SCANNING ELECTRON MICROSCOPY (SEM) 3
- CIRCULARLY POLARIZED LUMINESCENCE (CPL). 3

SUPPLEMENTARY TABLES AND FIGURES 4

SUPPLEMENTARY REFERENCES 14

Methods

Materials. All reagents were obtained from Sigma-Aldrich. Deionised water (18 MΩ cm resistivity) was obtained from a Direct-Q® 3 UV Water Purification System (Merck).

Preparation of 1. Ag-thioguanosine, **1**, prepared as follows. An aqueous suspension of 6-thioguanosine (6TG-H) was sonicated for 1 hour to achieve a fine dispersion to which was added a molar equivalent of $AgNO_3$ (solid). The reaction mixture was stirred for hour. As an example, a gel sample at concentration of 30 $mmol^{-1}$ was prepared by sonication of 18.0 mg of 6-thioguanosine in 2 ml of water for 1 hour. Then, the suspension was mixed with 10.2 mg of finely powdered $AgNO_3$. After the addition the mixture was stirred for an hour. Over the course of this period a yellow pale gel formed, which was indicated by the inverted-vial test (Fig. 1). There is no effect of stir direction has been notice on the gel (SI Fig. 6c).

Atomic Force Microscopy (AFM). AFM data was acquired using a Multimode 8 atomic force microscope with a NanoscopeV controller (Bruker), and a ''E'' scanner. Nanoscope software version 9.1 was used to control the microscope. The system was operated in ScanAsyst in Air mode as a peak force tapping mode at ultra-low forces minimise damage to the samples. For reducing vibrational noise, an isolation table/acoustic enclosure was used (Veeco Inc., Metrology Group). Silicon tips on silicon nitride cantilevers (ScanAsyst, Bruker) were used for imaging. The nominal tip radius was approximately 2 nm, resonant frequency 150 kHz and spring constant k ~0.7 Nm^{-1}. The AFM data were analysed with NanoScope Analysis 1.5 software (Bruker). The sample was prepared by adding 2µl of freshly prepared dilute aqueous solution of **1** onto a clean silicon wafer and drying in air.

Ultraviolet-Visible Spectroscopy (UV-Vis). Absorption spectra (in the range of 190-850 nm) were recorded in a NanoDrop™ One^C UV-Vis spectrophotometer. UV-Vis spectra of an aqueous solutions of 6-thioguanosine in at concentrations of 1 mmol l^{-1} and of Ag-thioguanosine (as an aqueous solution at concentrations of 1 mmol l^{-1} and as gel at concentration of 30 mmol l^{-1}) were recorded in a quartz cuvette with path length 0.1 mm. The spectrometer was blanked using Nanopure water.

FTIR spectroscopy. FTIR Spectra (in the range of 600 to 4000 cm^{-1} wavenumbers) were recorded using the ATR accessory of an IRAffinity-1S Fourier transform infrared spectrophotometer (Shimadzu) at 4 cm^{-1} spectral resolution. For each spectrum, 64 scans were co-added and averaged. The bare ATR accessory was used as a background. A sample of Ag-6TG was dried in air for 1 day prior to analysis and then deposited on a clean p-Si(100) chip (1 cm^2).

Matrix-assisted laser desorption/ionization Spectrometry (MALDI-Tof). MALDI spectrum was recorded on a Bruker Autoflex II ToF/Tof Mass Spectrometer. Ag-6TG hydrogel (60 mmol l^{-1}) was diluted in ultrapure water 100 times and 1µL was mixed in 9µL acetonitrile solution of α-cyano-4-hydroxycinnamic acid as a matrix.

Powder X-ray Diffraction (XRD). Very slow dehydration of the gel over 14 days yielded a fibrous green-yellow powder that exhibited a main Bragg diffraction ring at 3.03 Å.

Fluorescence spectroscopy. Emission spectroscopy (in the range 250-1000 nm) was recorded on a SPEX Fluoromax spectrofluorimeter. Emission spectra of solutions of 6-thioguanosine in 0.1 mol l^{-1} of NaOH (at concentrations of 1 mmol l^{-1} and 30 mmol l^{-1}) and of Ag-thioguanosine (as an aqueous solution at concentrations of 1 mmol l^{-1} and as gel at concentration of 30 mmol l^{-1}) were recorded in a quartz cuvette with pathlength 10 mm. The excitation wavelength was 350 nm for the solutions and 430 nm for the gel.

Circular dichroism. Circular dichroism spectra were recorded on a Jasco J-810 Jasco J-810 Spectropolarimeter. CD spectra of 6-thioguanosine solution (10 mmol l^{-1}) in 0.1 M of NaOH and Ag-thioguanosine as a gel (at concentration of 10 mmol l^{-1}) were recorded in a quartz cell with pathlength 0.1 mm.

Computational details. All TDDFT calculations were performed with the ORCA quantum chemistry package[1, 2] within the Tamm–Dancoff approximation.[3] All atoms were modelled with an all-electron Gaussian basis set of triple-ζ valence quality (def2TZVP)[4] along with the hybrid-level PBE0 exchange-correlation functional[5] and the semiempirical D3 Grimme dispersion correction.[6] The water solvent was simulated by employing the conductor–like polarizable continuum model.[7] Firstly, a geometry optimisation was performed on the crystal structure. From the optimised structure, we generated structures of increasing length, from 1 to 7 units - where one unit contains one silver atom and its associated organic neighbours. These unit structures were used to calculate all the excited states with a wavelength of 150 nm or more, along with the associated origin-independent velocity rotatory strengths, R_i.[8] We model the circular dichroism spectra, Δε, as the sum of Gaussian functions, each centred on the excitation energy E_i, whose amplitude is related to the rotatory strength and whose width, σ = 0:04E, is chosen empirically to best reproduce the experimental line shape,

$$\Delta\varepsilon = \frac{1}{k_R}\frac{1}{\sqrt{\pi}\sigma}\sum_i E_i\, R_i e^{-[(E-E_i)/\sigma]^2} \tag{1}$$

Rheology. Rheological measurements were performed with a HR-2 Discovery Hybrid Rheometer (TA Instruments) with a standard steel parallel-plate geometry of 20 mm diameter with a gap of 1 mm. The strain and the frequency were set to 1% and 1 Hz, respectively.

Scanning electron microscopy (SEM). Samples of xerogel Xe1 were dried on silicon wafers or freezing dried. The SEM images were collected using a TESCAN VEGA LMU Scanning Electron Microscope, housed within EM Research Services, Newcastle University. Digital images collected with TESCAN supplied software.

Circularly polarized luminescence (CPL). CPL spectra were recording using a custom-build CPL spectrometer. Full details of this CPL spectrometer have been reported by Carr et al.[9] Non-standard protocols were followed to measure CPL from the gel samples. Total emission and CPL emission were sampled at 400 - 800 nm in 5 nm increments, with 20 accumulated spectra per measurement. Roughly 500 micro-liters of the gel sample was contained in an open-topped quartz cuvette (101-10-40, Hellma). The gel adhered to a corner of the cuvette. Excitation was provided by a 410 nm laser source from directly above the sample. The laser and sample were positioned to maximise emission intensity from the sample. Data was processed using custom-written Matlab scripts (Matlab 2019b, Mathworks). Instrumental baselines for total emission intensity and CPL emission were subtracted to zero measurements as appropriate and were then smoothed using an intensity-preserving Savitzky-Golay filter. g_{lum} values for each repeated measurement were calculated from smoothed intensity and smoothed CPL emission. This was verified across 6 independent measurements. Quoted uncertainty is the standard deviation of g_{lum} of these six independent measurements. The uncertainty of the presented data is the standard deviation of 6 independent measurements.

Supplementary tables and figures

Supplementary Table 1. Wavelengths and rotatory strengths of the four excitations used to approximately reproduce the complete spectra. Also included is the angle between the electric and magnetic transition velocity dipoles.

State	ID	Wavelength (nm)	$R_i(10^{40}$cgs)	Angle (Deg)
**c**	569	199.6	-71.4	116.7
**d**	327	216.0	48.3	42.6
**e**	223	227.6	-113.5	173.0
**f**	22	297.6	67.8	76.0

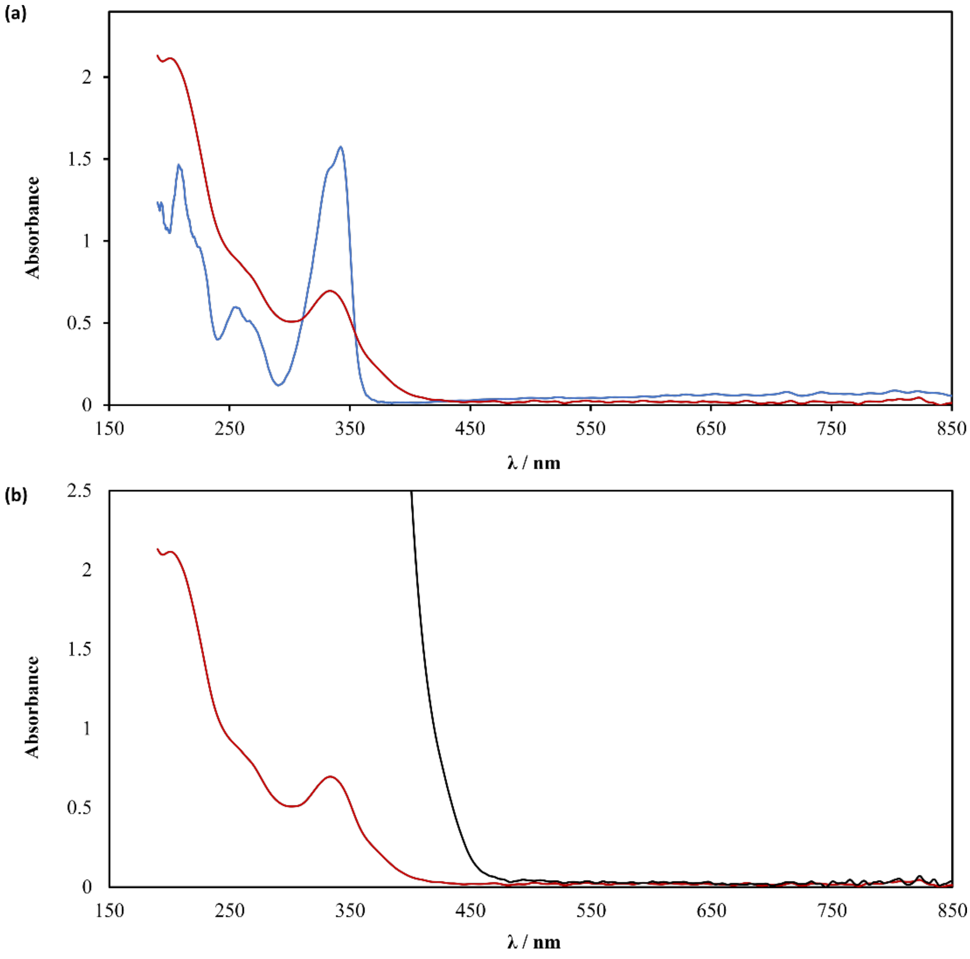

Supplementary Figure 1. (a) UV-Vis spectra of 6-TGH (blue) and of Ag-thioguanosine, **1**, (red), both in aqueous solution at a concentration of 1 mmol l^{-1}. (b) UV-Vis spectra of a solution of Ag-thioguanosine at a concentration of 1 mmol l^{-1} (red) and of a gel of Ag-thioguanosine at a concentration of 30 mmol l^{-1} (black). The samples were measured in a quartz cuvette with light-path of 0.1 mm.

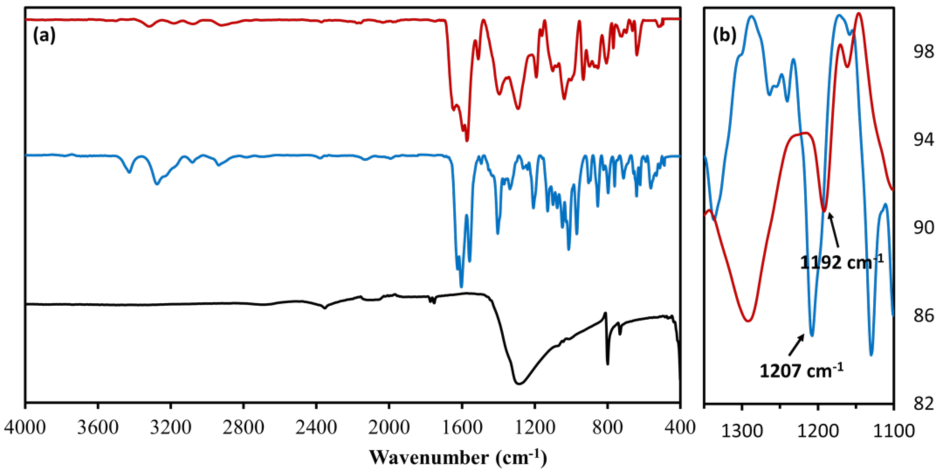

Supplementary Figure 2. (a) FTIR spectrum of Ag-thioguanosine gel (red), 6-thioguanosine (blue) and $AgNO_3$ (black). (b) Zoom in area in the spectra shows the region between 1100 and 1350 cm^{-1}.

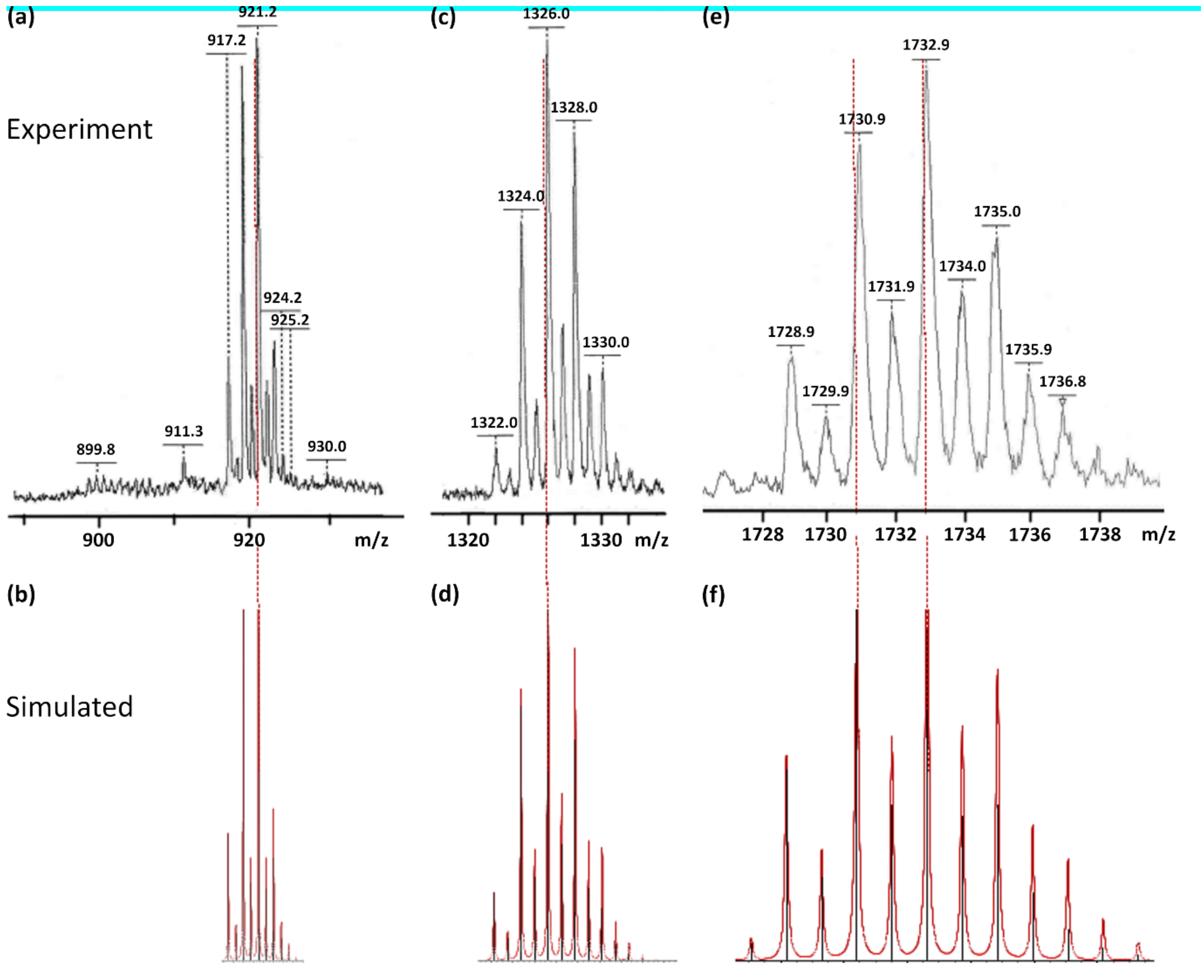

Supplementary Figure 3. MALDI data measured for Ag-thioguanosine. L = singly deprotonated form of thioguanosine, $[C_{10}H_{12}N_5O_4S]^-$. (a) $[Ag_3L_2]^+$ experimental, (b) $[Ag_3L_2]^+$ simulated; (c) $[Ag_4L_3]^+$ experimental, (d) $[Ag_4L_3]^+$ simulated and (e) $[Ag_5L_4]^+$ experimental, (f) $[Ag_5L_4]^+$ simulated. The red dotted lines show selected corresponding peaks in simulated and experimental spectra; the m/z values are tabulated below.

Species	Experimental m/z	Simulated m/z
$[Ag_3L_2]^+$	921.2	920.84
$[Ag_4L_3]^+$	1326.0	1325.80
$[Ag_5L_4]^+$	1730.9	1730.77
$[Ag_5L_4]^+$	1732.9	1732.77

Isotope pattern simulation using Gabedit v2.5.1.[10]

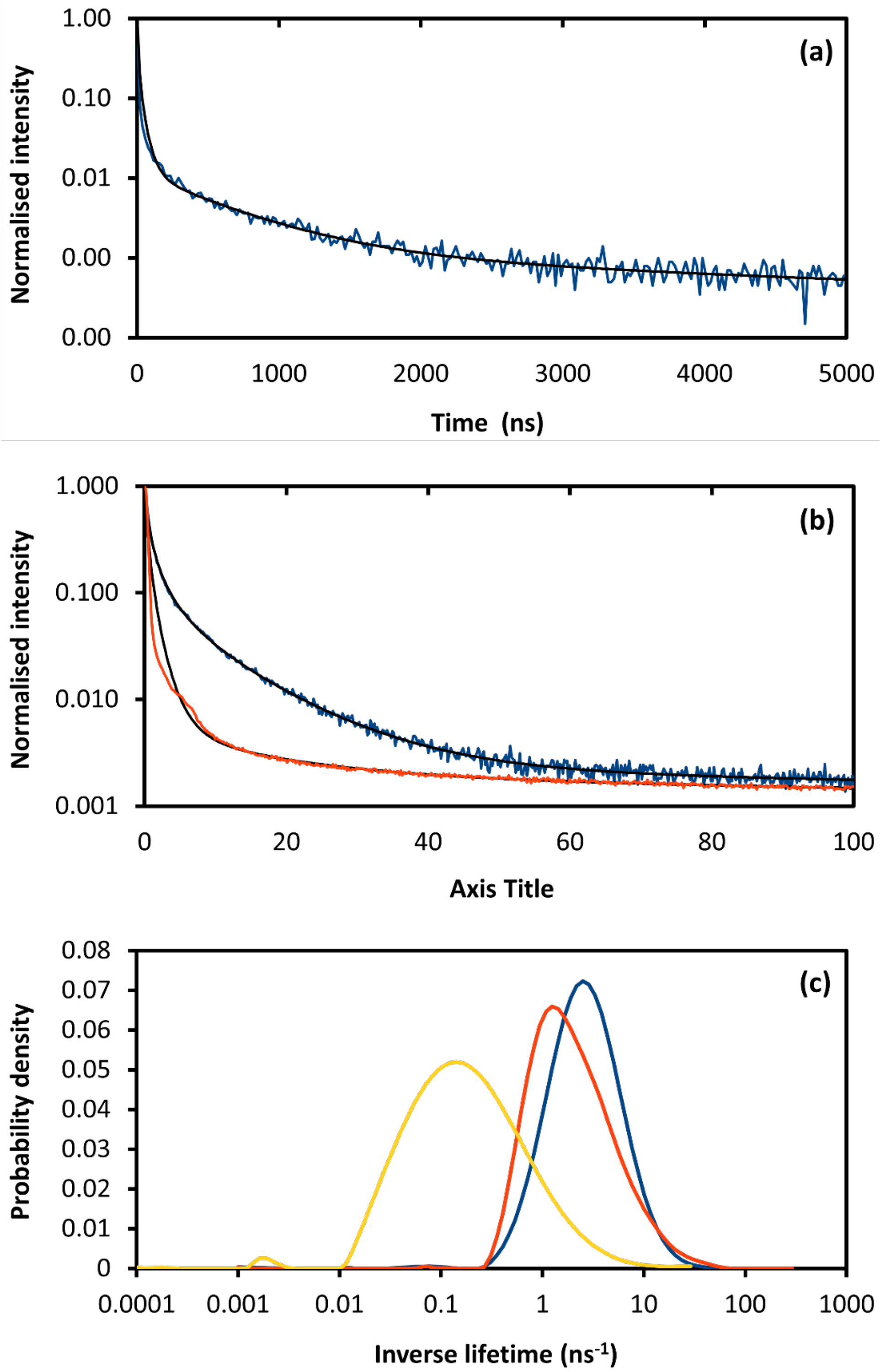

Supplementary Figure 4. (a) Normalised intensity against time for the luminescence decay of a gel of ***1*** at 30 mmol concentration and fit. The excitation wavelength was 371 nm and the emission at 510 nm was recorded against delay time in a TCSPC experiment with 10^6 counts. (b) Normalised intensity against time for the luminescence decay of ligand (6-thioguanosine, red line) and ***1*** (blue line) at 5 μ mol concentration and fits. The excitation wavelength was 371 nm and the emission at 430 nm was recorded against delay time in a TCSPC experiment with 10^6 counts. (c) Distribution of inverse lifetimes extracted from the fitting of the PL decays. blue curve - ligand; orange curve - 5 μ mol l^{-1} (**1**) and yellow curve (**1** gel) - 30 m mol l^{-1}. Note that a multiexponential fit is equivalent to approximating the distributions by delta functions.

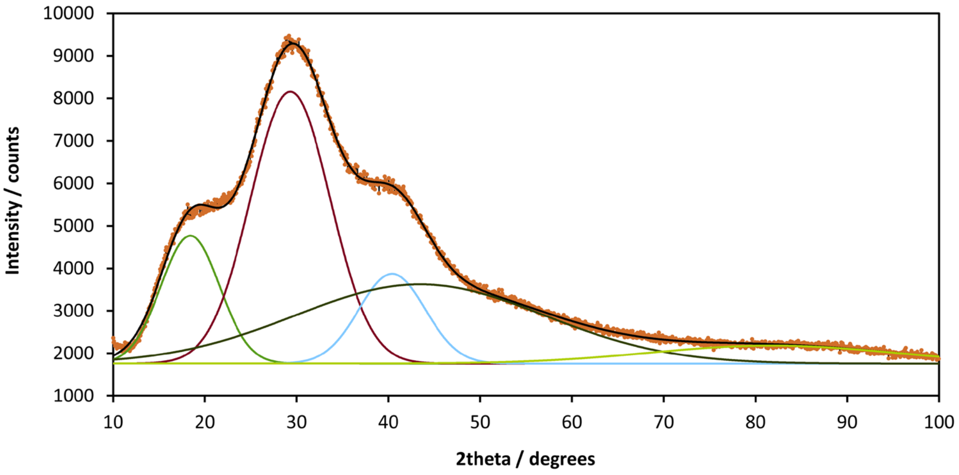

Supplementary Figure 5. Powder X-ray diffraction pattern for Ag-thioguanosine. The fitted regression model (black line) was a sum of 5 Gaussian functions and the coloured lines show the individual Gaussians. The peak at 2ϴ = 29.3 degrees can be interpreted as an Ag…Ag distance of 3.04 Å.

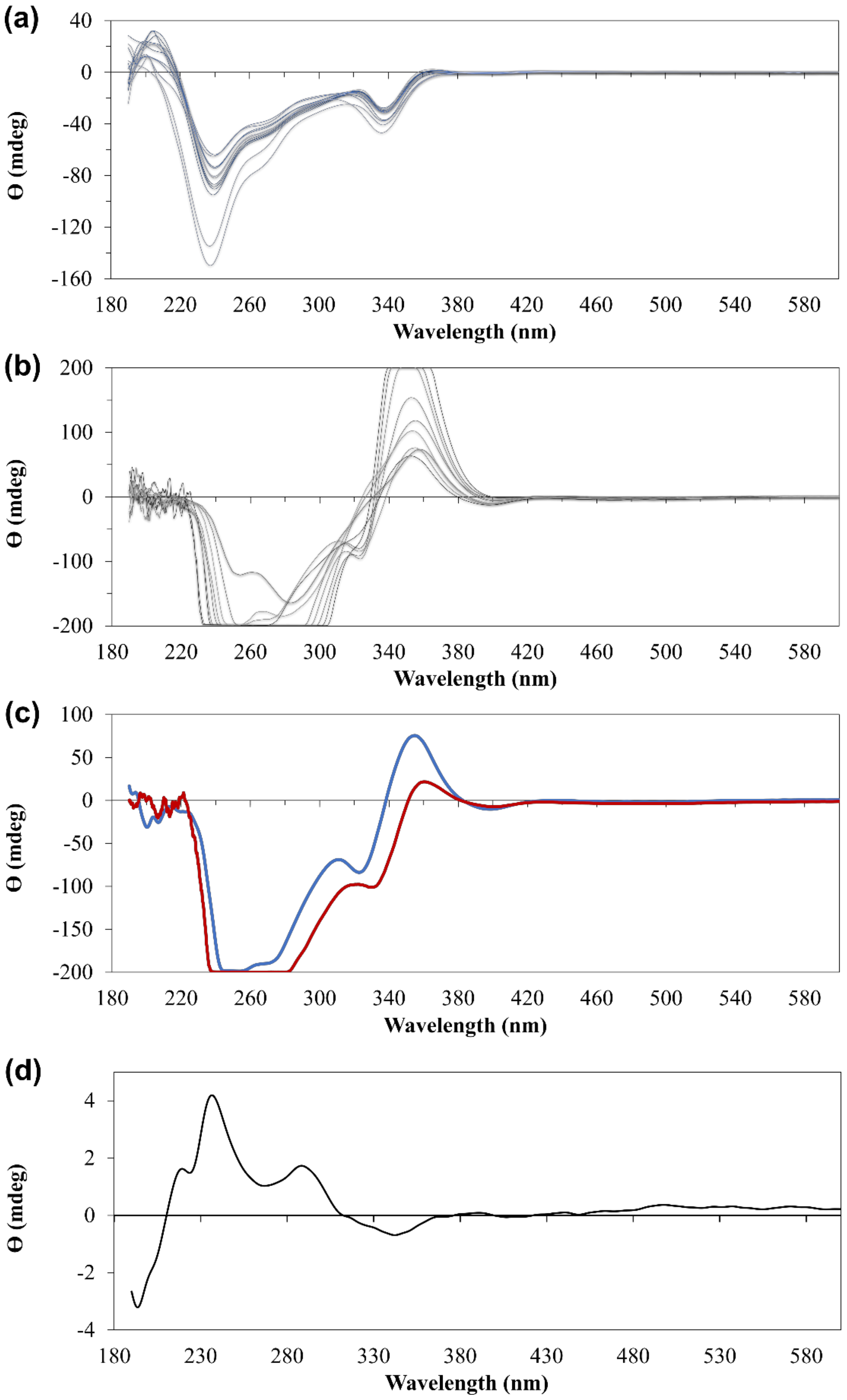

Supplementary Figure 6. CD spectra of Ag-thioguanosine, **1**, (a) solution at a concentration of 10 mmol l^{-1} and (b) a gel at concentration of 30 mmol l^{-1}. The CD spectra (a-b) show data from 12 replicated experiments. (c) CD spectra of a gel of Ag-thioguanosine at a concentration of 30 mmol l^{-1} formed with clockwise (red) and anticlockwise (blue) stirring. (d) CD spectrum of an aqueous solution of 6-TGH

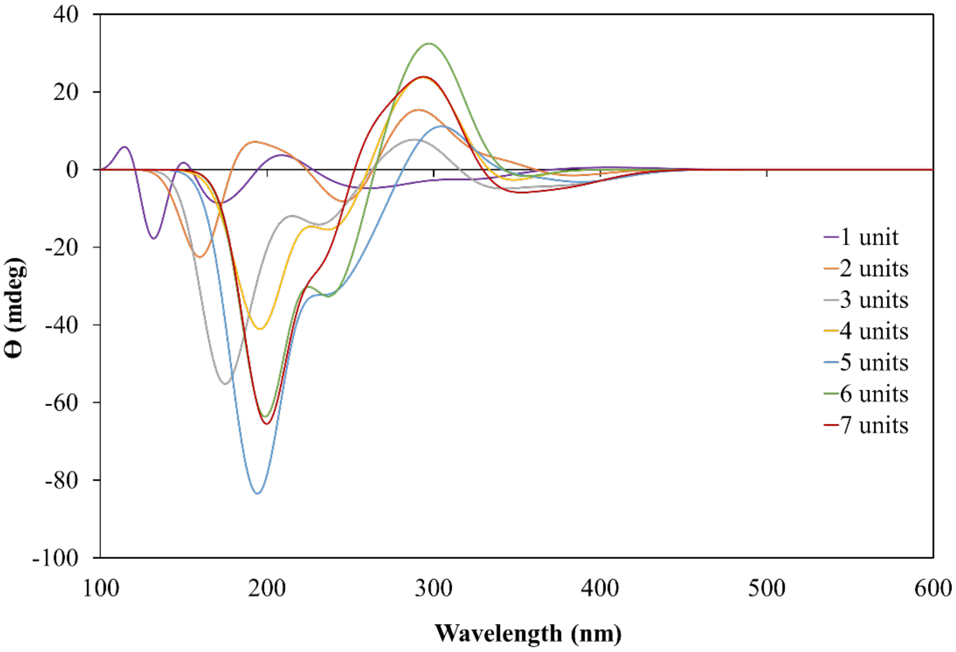

Supplementary Figure 7. Circular dichroism spectra calculated for 1-7 units of the Ag-thioguanosine structure.

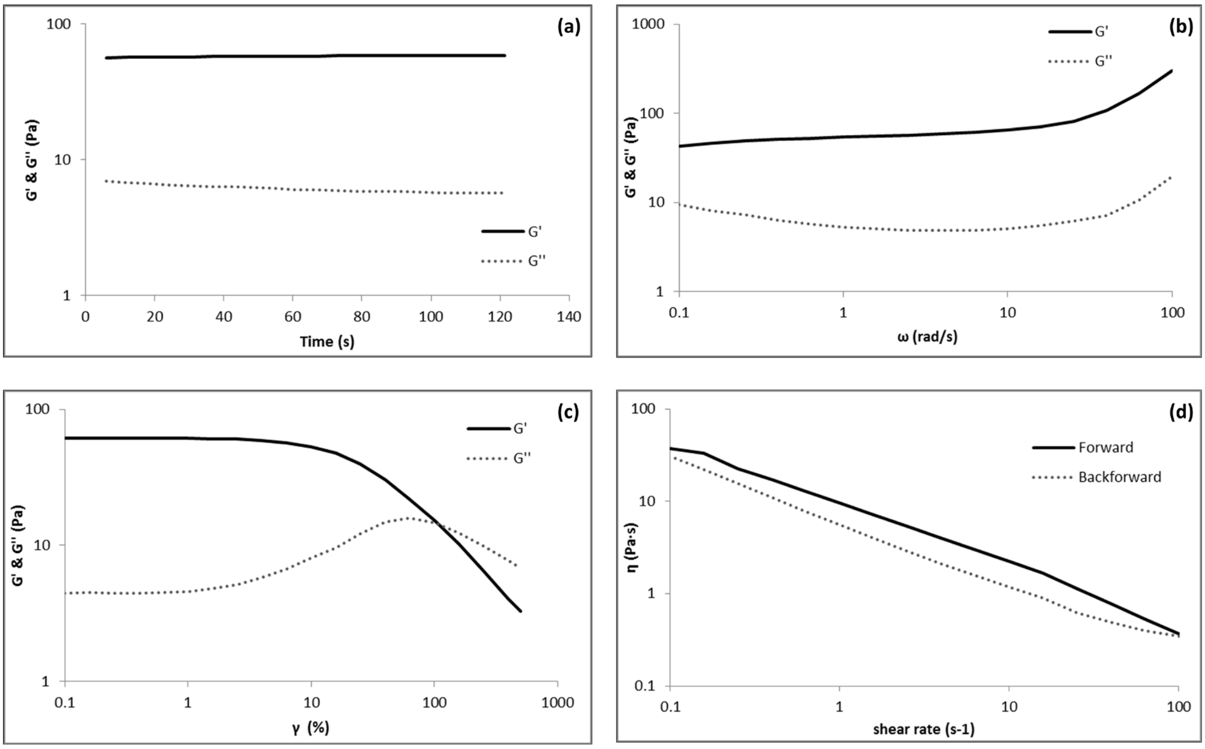

Supplementary Figure 8. Rheological characterisation of Ag-thioguanosine at 30 mmol l^{-1}. (a) Time sweep experiments. (b) Frequency sweep experiments. (c) Amplitude sweep experiments at 30 mmol l^{-1}. (d) Viscosity versus shear rate profile between 0.1 and 100 s^{-1}.

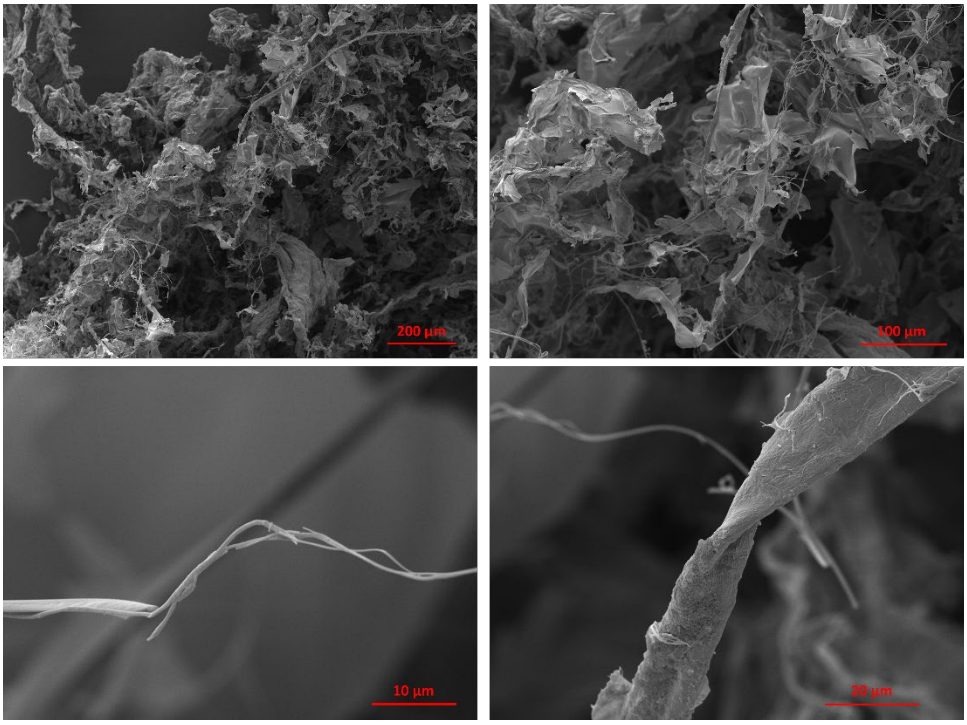

Supplementary Figure 9. SEM images of Ag-6TG at concentration of 6 mmol l^{-1} after the solution was freezing dried.

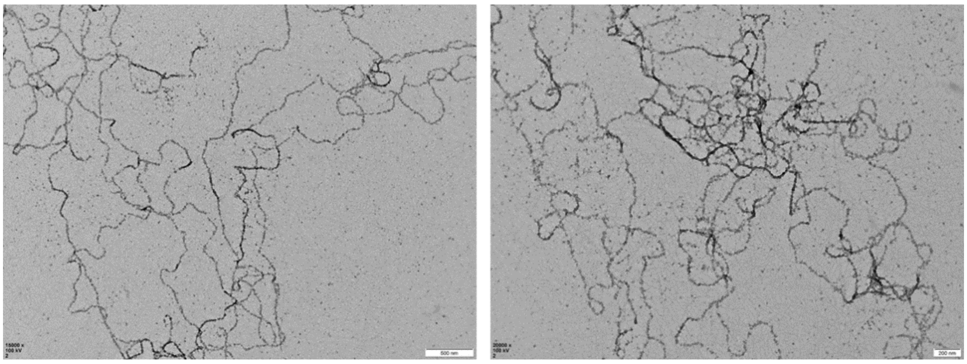

Supplementary Figure 10. TEM images of Ag:6-TG xerogel. 1 mmol l^{-1} Ag-thioguanosine solution was dried on continuous carbon coated grids.

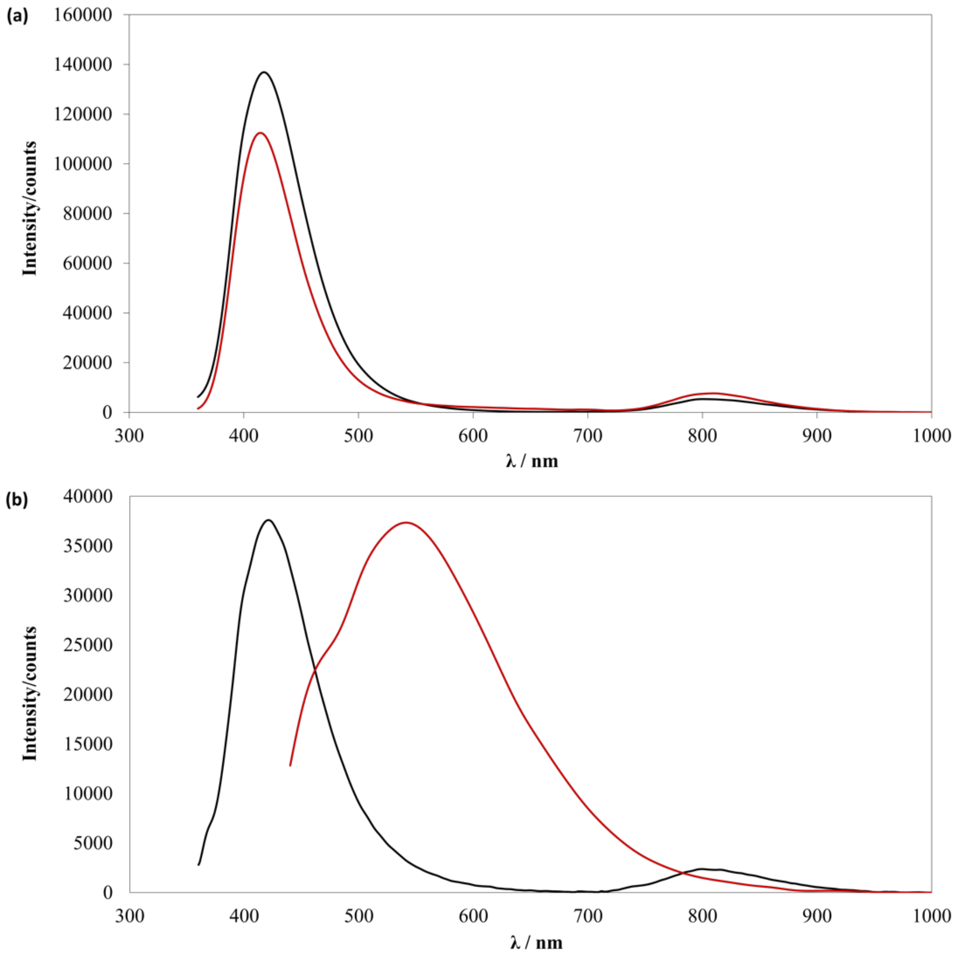

Supplementary Figure 11. (a) Fluorescence emission spectra of a solution of 6-TGH nucleoside in 0.1 mol l^{-1} of NaOH (black) and of an aqueous solution of Ag-thioguanosine (red), both at a concentration of 1 mmol l^{-1}. The excitation wavelength was 350 nm and the pathlength was 1 cm. (b) Fluorescence emission spectra of a solution of 6-TGH nucleoside in 0.1 mol l^{-1} of NaOH (black) and a gel of Ag-thioguanosine (red), both at a concentration of 30 mmol l^{-1}. The excitation wavelength was 350 nm for the 6-TGH solution and 430 nm for the Ag-thioguanosine gel and the pathlength was 10 mm.

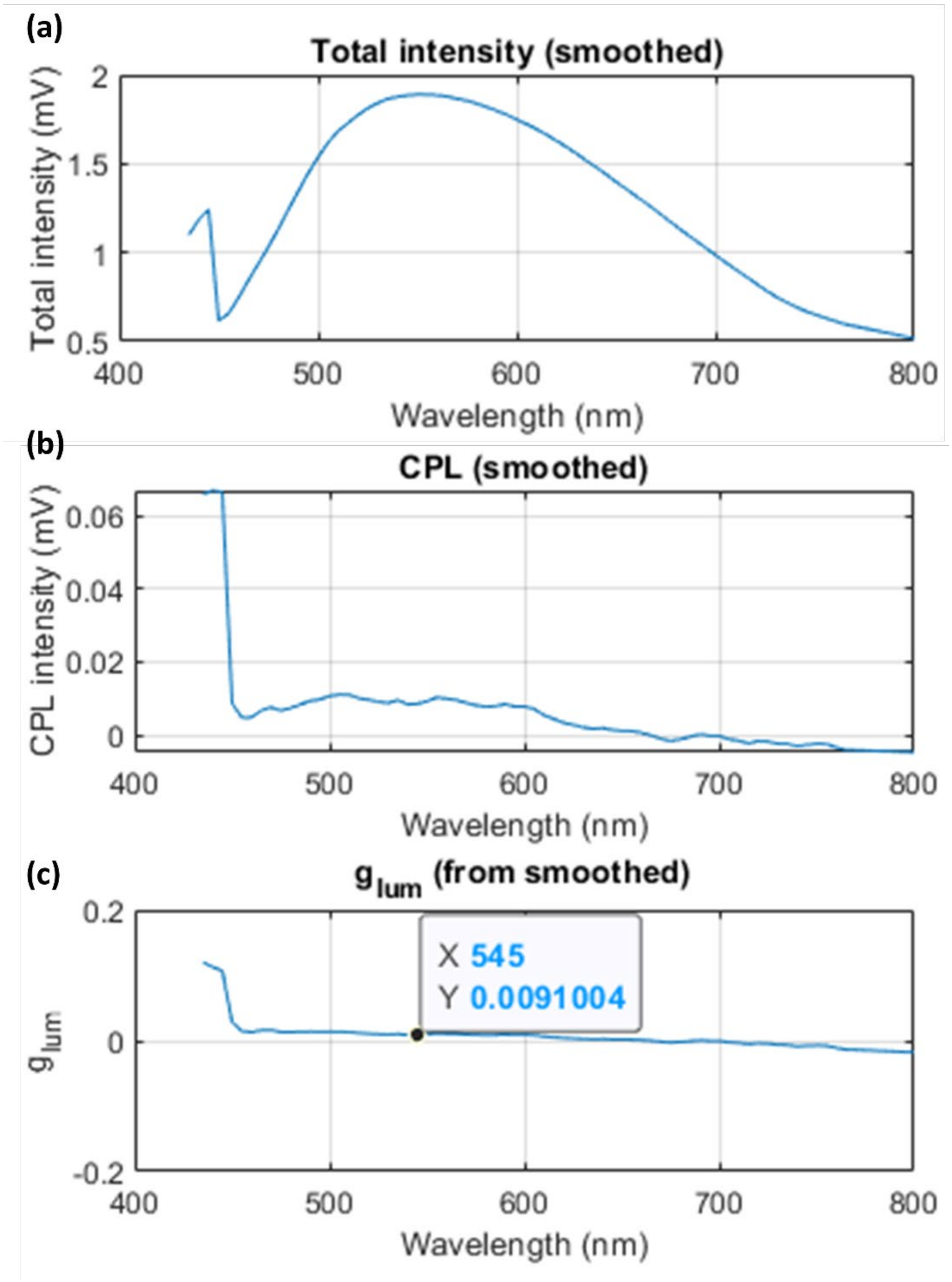

Supplementary Figure 12. (a) Total photoluminescence spectrum of Ag-thioguanosine solution at concentration of 10 mmol l^{-1}. (b) CPL smoothed spectrum of the Ag-thioguanosine solution. (c) The luminescence dissymmetry factor (**g_{lum}**) data from the smoothed spectrum of the Ag-thioguanosine shows a g_{lum} value of about 0.01. The excitation wavelength was 410 nm, which can be seen as an artefact in CPL and g_{lum} spectra.

Supplementary references

1 F. Neese, *Wiley Interdiscip. Rev. Comput. Mol. Sci.* , 2012, **2**, 73-78.
2 F. Neese, *Wiley Interdiscip. Rev. Comput. Mol. Sci.* , 2018, **8**, 1327.
3 S. Hirata and M. Head-Gordon, *Chem. Phys. Lett.*, 1999, **314**, 291-299.
4 F. Weigend and R. Ahlrichs, *Phys. Chem. Chem. Phys.*, 2005, **7**, 3297-3305.
5 C. Adamo and V. Barone, *J. Chem. Phys.*, 1999, **110**, 6158-6170.
6 S. Grimme, *J. Comput. Chem.*, 2006, **27**, 1787-1799.
7 D. M. York and M. Karplus, *J. Phys. Chem. A*, 1999, **103**, 11060-11079.
8 S. Grimme, F. Furche and R. Ahlrichs, *Chem. Phys. Lett.*, 2002, **361**, 321-328.
9 R. Carr, R. Puckrin, B. K. McMahon, R. Pal, D. Parker and L. O. Palsson, *Methods Appl. Fluoresc.*, 2014, **2**.
10 A. R. Allouche, *J. Comp. Chem.*, 2011, **32**, 174–182.
